# Notch signaling deficiency underlies age-dependent depletion of satellite cells in muscular dystrophy

**DOI:** 10.1242/dmm.015917

**Published:** 2014-06-06

**Authors:** Chunhui Jiang, Yefei Wen, Kazuki Kuroda, Kevin Hannon, Michael A. Rudnicki, Shihuan Kuang

**Affiliations:** 1 Department of Animal Sciences, Purdue University, West Lafayette, IN 47907, USA; 2 Molecular Medicine Program, Ottawa Hospital Research Institute, Ottawa, ON K1Y 4E9, Canada; 3 Department of Basic Medical Sciences, Purdue University, West Lafayette, IN 47907, USA

**Keywords:** Muscular dystrophy, Notch signaling, Stem cell

## Abstract

Duchenne muscular dystrophy (DMD) is a devastating disease characterized by muscle wasting, loss of mobility and death in early adulthood. Satellite cells are muscle-resident stem cells responsible for the repair and regeneration of damaged muscles. One pathological feature of DMD is the progressive depletion of satellite cells, leading to the failure of muscle repair. Here, we attempted to explore the molecular mechanisms underlying satellite cell ablation in the dystrophin mutant *mdx* mouse, a well-established model for DMD. Initial muscle degeneration activates satellite cells, resulting in increased satellite cell number in young *mdx* mice. This is followed by rapid loss of satellite cells with age due to the reduced self-renewal ability of *mdx* satellite cells. In addition, satellite cell composition is altered even in young *mdx* mice, with significant reductions in the abundance of non-committed (Pax7^+^ and Myf5^−^) satellite cells. Using a Notch-reporter mouse, we found that the *mdx* satellite cells have reduced activation of Notch signaling, which has been shown to be necessary to maintain satellite cell quiescence and self-renewal. Concomitantly, the expression of *Notch1*, *Notch3*, *Jag1*, *Hey1* and *HeyL* are reduced in the *mdx* primary myoblast. Finally, we established a mouse model to constitutively activate Notch signaling in satellite cells, and show that Notch activation is sufficient to rescue the self-renewal deficiencies of *mdx* satellite cells. These results demonstrate that Notch signaling is essential for maintaining the satellite cell pool and that its deficiency leads to depletion of satellite cells in DMD.

## INTRODUCTION

Muscular dystrophies include a spectrum of inherited diseases that lead to progressive muscle degeneration and dysfunction (Wallace and McNally, 2009). The most severe and common form of muscular dystrophy is Duchenne muscular dystrophy (DMD). DMD is a devastating recessive X-linked muscle degenerative disease caused by frame-shift deletions, duplications, or point mutations in the dystrophin (*DMD*) gene (Worton et al., 1984; Hoffman et al., 1987). Dystrophin is a cytoskeletal protein that interacts with a group of peripheral membrane and transmembrane proteins, such as dystroglycan and sarcoglycan, to form the dystrophin-associated protein complex (DAPC) (Matsumura et al., 1994). The DAPC provides a link between the cytoskeleton and extracellular matrix of muscle fibers, and maintains the integrity of sarcolemma (muscle membrane) during muscle contraction (Ervasti and Sonnemann, 2008). Absence of dystrophin results in the disassociation of the DAPC. As a consequence, the sarcolemma becomes fragile to mechanical damage, and normal muscle activity can result in muscle degeneration, chronic inflammation and fibrosis (Petrof et al., 1993). These pathological stimulations alter the tissue environment and compromise muscle function to further deteriorate the dystrophic phenotype. DMD patients typically suffer from rapid progression of muscle degeneration, and are eventually paralyzed and die in their second to third decade of life.

Skeletal muscles have a remarkable capacity to regenerate. This capacity is mainly attributed to a stem cell population called satellite cells. Satellite cells are muscle-specific adult stem cells that are responsible for muscle regeneration in response to injuries (Chargé and Rudnicki, 2004). Upon a muscle injury, satellite cells, which are located between the basal lamina (i.e. the muscle extracellular matrix) and sarcolemma, are activated from quiescence and proliferate as myogenic precursor cells; the proliferating myoblasts then undergo either self-renewal and return to quiescence, or differentiation to form functional muscles (Wagers and Conboy, 2005; Anderson, 2006; Kuang and Rudnicki, 2008; Cheung and Rando, 2013). In healthy humans, satellite cells can proliferate and repair muscle damage. However, the unrelenting muscle degeneration in DMD puts satellite cells in a constant activation mode and eventually depletes the satellite cell pool, leading to the failure of muscle repair and accelerated disease progression (Blau et al., 1983; Blau et al., 1985; Heslop et al., 2000).

Currently, there is no effective treatment for DMD patients. To date, stem-cell-based therapeutic strategies are under intense investigation. Such stem cell therapies mainly include delivery of exogenous muscle stem cells to boost the regeneration of DMD muscles and functional enhancement of endogenous muscle stem cells (Shi and Garry, 2006; Bentzinger et al., 2010; Quattrocelli et al., 2010). However, stem cell therapies are still in their infancy and, to achieve the full potential of these regenerative approaches, it is necessary to better understand the cellular and molecular mechanisms governing satellite cell behavior and function. Previous studies have shown that the Notch signaling pathway plays important roles in maintaining satellite cell quiescence, as well as regulating proliferation and differentiation (Conboy and Rando, 2002; Buas and Kadesch, 2010; Bjornson et al., 2012; Mourikis et al., 2012). Constitutive activation of Notch pathway promotes the self-renewal of satellite cells by upregulating Pax7, a key regulator of satellite cell identity (Wen et al., 2012). Conversely, blockage of Notch signaling in satellite cells results in muscular dystrophy characteristics and impairs muscle regeneration (Lin et al., 2013). However, whether Notch signaling is deregulated in the satellite cells of dystrophic muscles and whether it contributes to the progression of muscle degeneration have not been determined.

**TRANSLATIONAL IMPACT**

**Clinical issue**

Muscle wasting conditions compromise mobility and quality of life, and can eventually prove fatal. Muscle wasting is common in the elderly (ageing-associated muscle degeneration is known as sarcopenia), in cancer patients (known as cachexia) and in degenerative muscle diseases such as Duchenne muscular dystrophy (DMD). DMD, a recessive X-linked disorder that affects about 1 in 3500 male births is the most severe muscle wasting disease. Currently there is no cure for DMD. The inability of the body to repair the diseased muscle in DMD patients is due to progressive depletion of satellite cells, a stem cell population that normally helps to regenerate injured muscles in healthy individuals. In this study, the authors investigate the molecular mechanisms underlying satellite cell ablation in an established mouse model of DMD, the *mdx* mouse.

**Results**

The authors report that satellite cells can be activated normally to repair muscle injuries in young *mdx* mice. Satellite cell number was observed to decrease with age: 6-month-old mice demonstrated a rapid loss of satellite cells. These mice are equivalent to 20-year-old humans affected with DMD; usually, this is the stage at which immobility occurs. The ability of satellite cells to respond to injury also rapidly declined with age in the *mdx* mice. The age-dependent decline in the satellite cell number and activity was found to be correlated to impairments in Notch signaling – an evolutionary conserved signaling cascade that has previously been implicated in muscle stem cell function. Interestingly, the authors show, by using another mouse model, that deficits in satellite cell activity can be restored in *mdx* mice by artificially switching on Notch signaling.

**Implications and future directions**

This study provides evidence that satellite cell numbers decline with age and their self-renewal capacity is impaired in *mdx* mice, in line with the important role of this stem cell population in muscle regeneration. Perturbation of the Notch signaling pathway is shown to be linked to depletion of satellite cells in diseased mice, indicating that Notch signaling is essential for maintaining the satellite cell pool. Restoration of the Notch signaling pathway appears to restore the self-renewal capacity of *mdx* satellite cells. This finding points to the possibility of using pharmacological compounds to activate Notch signaling to prevent satellite cell loss and preserve satellite cell functions in DMD patients.

In this study, we aimed to address these questions by using the *mdx* mouse model (Bulfield et al., 1984), which carries a mutation in the *Dmd* gene and thus has been widely used as an animal model for human DMD (Partridge, 2013). We discovered that *mdx* satellite cells exhibit defective self-renewal capacity associated with attenuated Notch signaling transduction. Importantly, constitutive activation of Notch signaling in the *mdx* satellite cells rescued their self-renewal defects. These data demonstrate that the attenuated Notch signaling in *mdx* mice leads to satellite cell dysfunction and further suggest that Notch signaling has the therapeutic potential to retain the self-renewal capacity in dystrophic muscles.

## RESULTS

### Satellite cell number and activity decline with age in *mdx* mice

**Fig. 1.**
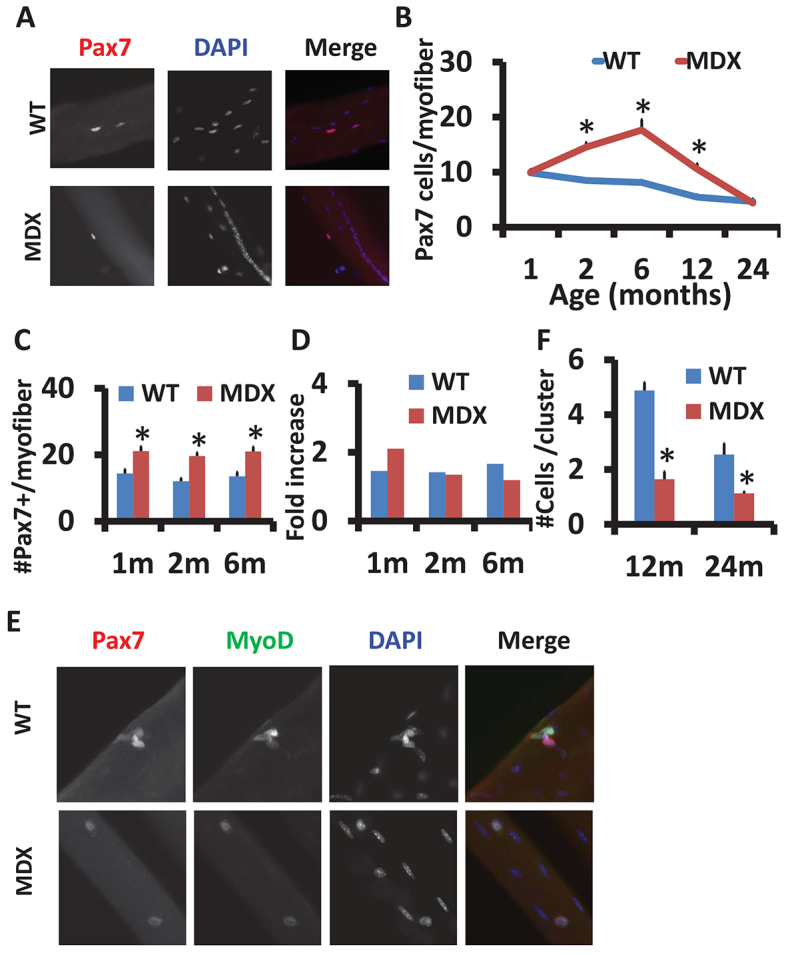
**Decline of satellite cells number and activity with age in *mdx* mice.** (A) Representative images of satellite cells in single EDL myofibers from WT and *mdx* mice, labeled with Pax7 (red). Nuclei were counterstained with DAPI (blue). Central nuclei indicate muscle regeneration in *mdx* mice. (B) The mean±s.e.m. satellite cell number per EDL myofiber from WT and *mdx* mice at different ages is shown (*n*=3 mice with more than 20 fibers analyzed in each mouse at each indicated age). (C) Mean±s.e.m. satellite cell number per EDL myofiber for WT and *mdx* mice after CTX treatment at the indicated ages (*n*=3). (D) The CTX-stimulated relative fold increase of satellite cells calculated from data in B and C. (E) Representative images of satellite cell clusters on cultured (72 hours) single myofibers isolated from 24-month-old WT and *mdx* mice, labeled with Pax7 (red) and MyoD (green). Nuclei were counterstained with DAPI (blue). (F) Quantification (mean±s.e.m.) of satellite cell number per cluster based on data in E (*n*=3 independent experiments with more than 20 clusters analyzed in each experiment). *P<0.05 compared with WT.

As satellite cells are necessary for postnatal muscle regeneration (Lepper et al., 2011; Murphy et al., 2011; Sambasivan et al., 2011b), we aimed to examine satellite cell behavior in *mdx* mice, where muscles are under repetitive degeneration and regeneration. We first examined the abundance of satellite cells associated with freshly isolated myofibers from the extensor digitorum longus (EDL) muscles of wild-type (WT) and *mdx* mice at different ages (Fig. 1A). Interestingly, there were significantly more Pax7^+^ satellite cells per myofiber in 2-, 6- and 12-month-old *mdx* mice than in WT mice of the same age (Fig. 1B). Whereas the number of WT satellite cells continually declined with age, at a slow rate, the *mdx* satellite cell number initially increased in myofibers from 1-month- to 6-month-old mice, followed by a rapid decline afterwards (Fig. 1B). As the severity of the muscle pathology increases at ~2 months (Bulfield et al., 1984), the initial increases in satellite cell number reflect the activation of satellite cells due to ongoing muscle injuries. The rapid decline of satellite cell number starting at 6 months suggests that the *mdx* satellite cells are unable to maintain a proper balance of proliferation, self-renewal and differentiation.

We further examined the proliferative activity of satellite cells *in vivo* and *in vitro*. In response to cardiotoxin (CTX)-induced muscle degeneration, satellite cells were activated, and they proliferated, then fused to repair the injury. At 5 days post CTX injection into tibialis anterior (TA) muscles, the number of satellite cells per myofiber increased in both WT and *mdx* mice at 1–6 months of age (Fig. 1C). However, the CTX-stimulated increases of satellite cells rapidly decreased with age in the *mdx*, but not in the WT, mice (Fig. 1D). These results indicate that there are severe age-dependent deficiencies in the activation and/or proliferation of satellite cells in the *mdx* mice. We also cultured satellite cells while they were still attached onto their host myofibers, which were individually dissociated from 12- and 24-month-old mice (Fig. 1E). After 72 hours in culture, the WT satellite cells proliferated and formed clusters of cells, but the *mdx* satellite cells in both 12- and 24-month-old mice failed to form cell clusters (Fig. 1F). This observation confirms that satellite cell activity declines dramatically in aged *mdx* mice.

### *mdx* satellite cells have reduced self-renewal capacity

**Fig. 2.**
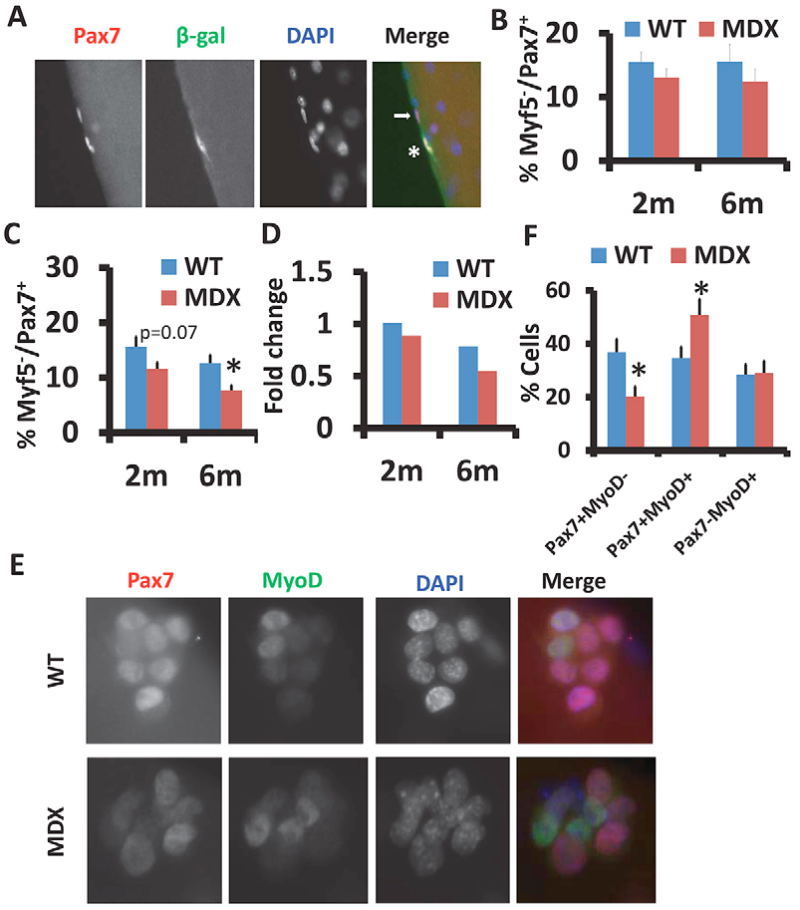
**Reduced self-renewal capacity of satellite cells in *mdx* muscles.** (A) Representative images of Pax7 (red) and β-gal (green) staining of single myofibers isolated from EDL muscles of *Myf5^nLacz^/mdx* mice. Pax7^+^β-gal^+^ (asterisk) and Pax7^+^β-gal^−^ (arrow) satellite cells are shown. Nuclei were counterstained with DAPI (blue). (B,C) Percentages of self-renewing [Pax7^+^Myf5^−^ (β-gal^−^)] satellite cells without CTX treatment (B) and with CTX treatment (C) at the indicated ages. Results are mean±s.e.m. (*n*=3 mice with more than 20 fibers analyzed in each mouse). (D) The CTX-stimulated relative fold increase of the percentages of self-renewing satellite cells calculated from data in B and C. (E) Representative images of satellite cell clusters from WT and *mdx* EDL muscle fibers labeled with Pax7 (red) and MyoD (green). Nuclei were counterstained with DAPI (blue). (F) Percentages of quiescent (Pax7^+^MyoD^−^), proliferating (Pax7^+^MyoD^+^) and differentiating (Pax7^−^MyoD^+^) cells. Data are mean±s.e.m. for three independent experiments with at least 20 clusters analyzed in each experiment. *P<0.05 compared with WT.

Self-renewal is a defining feature of all stem cells and is necessary for maintenance of stem cell homeostasis. We hypothesized that the age-dependent depletion of satellite cells in the *mdx* mice was due to a reduced self-renewal capacity. To test this hypothesis, we established the *Myf5^nLacZ^/mdx* mice by breeding *mdx* mice with *Myf5^nLacZ^* mice, which express nuclear-localized β-galactosidase in Myf5-expressing cells (Christov et al., 2007). Previous studies have shown that Pax7^+^Myf5^−^ satellite cells give rise to Pax7^+^Myf5^+^ satellite cells during myogenic commitment, and the Pax7^+^Myf5^−^ cells have higher self-renewal capacity (Kuang et al., 2007). We isolated EDL myofibers from WT (*Myf5^nLacZ/+^*) and *mdx* (*Myf5^nLacZ/+^/mdx*) mice that were injected with CTX to assure satellite cells in both WT and *mdx* mice were in the same activated state. Satellite cells were then labeled with antibodies to Pax7 and β-gal (Fig. 2A). In the absence of CTX-induced injury, similar proportions of Pax7^+^Myf5^−^ (β-gal^−^) satellite cells were found between WT and *mdx* mice, at both 2 and 6 months of age (Fig. 2B). After CTX-induced muscle injury, the abundance of Pax7^+^Myf5^−^ satellite cells was reduced in the *mdx* mice at both 2 and 6 months of age (Fig. 2C). Furthermore, the CTX-induced fold change of Pax7^+^Myf5^−^ satellite cells was decreased in *mdx* compared to in WT mice at both 2 and 6 months of age (Fig. 2D). These *in vivo* data demonstrate that there is a self-renewal defect in *mdx* satellite cells.

We further examined satellite cell self-renewal using a well-established paradigm involving culture of dissociated myofibers (Halevy et al., 2004; Olguin and Olwin, 2004; Zammit et al., 2004). After culture, the self-renewal, proliferation and differentiation progenies can be distinguished as Pax7^+^MyoD^−^, Pax7^+^MyoD^+^ and Pax7^−^MyoD^+^, respectively, based on their Pax7 and MyoD expression pattern (Fig. 2E). Quantitative analysis indicates that the percentage of Pax7^+^MyoD^−^ (self-renewing) cells was drastically decreased in *mdx* myofiber cultures (37% in WT vs 20% in *mdx*), whereas the percentage of Pax7^+^MyoD^+^ (proliferating) cells was increased in the *mdx* cultures, and the percentage of Pax7^−^MyoD^+^ (differentiating) cells was not different between WT and *mdx* cultures (Fig. 2F). Moreover, the expression of MyoG, a terminal differentiation marker of myogenesis, was examined after 72 hours of myofiber culture (supplementary material Fig. S2A). Quantitative analysis revealed that the ratio of MyoG^+^:MyoD^+^ cells was elevated in *mdx* myofibers from mice at both 2 and 6 months of age (supplementary material Fig. S2B), suggesting that *mdx* satellite cells have a higher tendency for terminal differentiation. Collectively, these cell culture data are consistent with the notion that the *mdx* satellite cells have reduced self-renewal capacity.

### The Notch signaling pathway is perturbed in *mdx* satellite cells

**Fig. 3.**
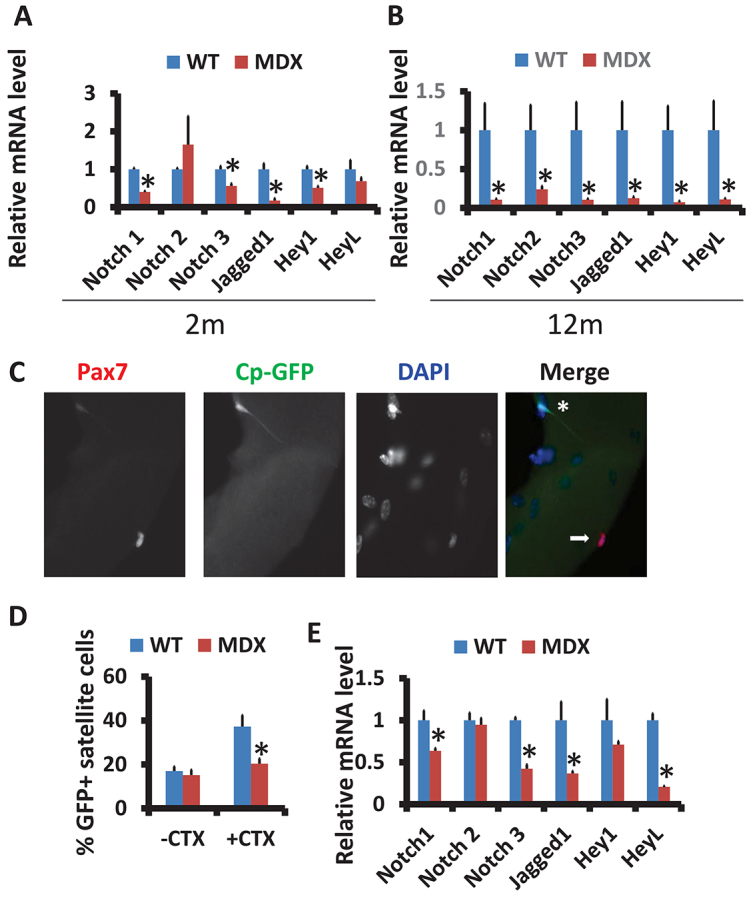
**Aberrant Notch signaling in the *mdx* satellite cells.** (A,B) Expression of genes related to the Notch signaling pathway in both young (2 months; A) and aged (12 months; B) *mdx* muscles (mean±s.e.m., *n*=6 mice). (C) Representative images of Pax7^+^GFP^+^ (asterisk) and Pax7^+^GFP^−^ (arrow) satellite cells on a single EDL myofiber isolated from Cp-GFP/*mdx* mice. Nuclei were counterstained with DAPI (blue). (D) Percentages of satellite cells with active Notch signaling (Pax7^+^GFP^+^) without and with CTX treatment (mean±s.e.m., *n*=3 mice with more than 20 fibers analyzed in each mouse). (E) WT and *mdx* primary myoblasts were cultured and collected for qPCR analysis for genes encoding Notch receptors (Notch1, Notch2 and Notch3) and ligand (Jagged 1) as well as those encoding Notch target genes (Hey1 and HeyL). Results are mean±s.e.m. for three independent experiments. *P<0.05 compared with WT.

To understand the molecular mechanism underlying the reduced self-renewal capacity in the *mdx* satellite cells, we investigated the Notch signaling pathway, which has been shown to mediate satellite cell self-renewal and quiescence (Vasyutina et al., 2007). We detected that the expression of genes related to Notch signaling pathway was reduced dramatically in both young (2 months; Fig. 3A) and old (12 months; Fig. 3B) *mdx* muscles. Strikingly, the reduction was much more robust in the old *mdx* mice (Fig. 3B compared to 3A). The reduced expression of Notch receptors, ligands and target genes in *mdx* muscles indicate an impairment of Notch signaling transduction.

To directly visualize the activation status of Notch signaling in satellite cells, we used the Cp-GFP reporter mouse (Mizutani et al., 2007). In this transgenic mouse, GFP expression is driven by four tandem repeats of a DNA sequence that is recognized by Rbpjk, the nuclear mediator of Notch signaling. When Notch is not activated, Rbpjk binds to transcriptional repressors that suppress GFP expression. Upon activation (ligand binding), the Notch intracellular domain (NICD) detaches and translocates to the nucleus, where it replaces the transcriptional repressors on Rbpjk and activates GFP expression. To establish the utility of this model in satellite cells, we first isolated single myofibers from the Cp-GFP mice and examined GFP and Pax7 expression (supplementary material Fig. S1A). A fraction (~17%) of Pax7^+^ satellite cells was also GFP^+^ (supplementary material Fig. S1B). Next, fluorescence-activated cell sorting (FACS) was used to isolated GFP^+^ and GFP^−^ satellite cells using α7 integrin (Int-α7) as a positive selection marker for satellite cells (supplementary material Fig. S1C). Analysis of FACS-purified satellite cells indicates that 8% of satellite cells (Lin^−^Int-α7^+^) were also GFP^+^ (supplementary material Fig. S1C). Importantly, the FACS-purified GFP^+^ satellite cells expressed higher levels of *Hes1* (supplementary material Fig. S1D), a canonical target of the Notch signaling pathway. To further examine whether the Cp-GFP reporter responds to Notch activation *in vivo*, we electroporated DNA plasmids encoding a Notch ligand (Jag1) or activated Notch (NICD, also called the RAMIC domain) into the TA muscles and analyzed Pax7 and GFP expression 7 days later (supplementary material Fig. S1E,F). Overall, Jag1 overexpression increased the percentage of satellite cells that were both Pax7^+^ and GFP^+^ by ~30% (*n*=1191 cells analyzed) and NICD overexpression increased the double-positive satellite cells by ~50% (*n*=825 cells analyzed). Thus, the Cp-GFP reporter mouse faithfully reports Notch signaling activation in satellite cells. The single-fiber and FACS analyses further demonstrate that Notch signaling is activated in a small population of quiescent satellite cells.

We next established the *Cp-GFP/mdx* mouse model through crossing the two lines of mice and examined Cp-GFP expression. EDL myofibers were isolated from WT control (*Cp-GFP*) and *mdx* (*Cp-GFP/mdx*) mice 5 days after CTX-induced regeneration, and labeled with Pax7 and GFP antibodies (Fig. 3C). Notably, the percentage of GFP^+^ satellite cells (GFP^+^Pax7^+^/total Pax7^+^) from *mdx* mice was only about half of that in WT mice after CTX injury (Fig. 3D; 37% in WT vs 20% in *mdx*). By contrast, there was no significant difference in the percentage of GFP^+^ satellite cells between WT and *mdx* mice under resting conditions (Fig. 3D). This result suggests that *mdx* satellite cells have reduced activation of Notch signaling during muscle regeneration. Consistent with this notion, the mRNA levels of the Notch receptors (*Notch1* and *Notch3*) and Notch ligand (*Jag1*) as well as that of a Notch target (*HeyL*) were reduced by ~50% in primary myoblasts derived from *mdx* mice compared to those from WT mice (Fig. 3E). Taken together, the self-renewal defects of *mdx* satellite cells is associated with reduced Notch signaling transduction.

### Constitutive activation of Notch rescues the self-renewal defects of satellite cells but fails to improve muscle pathology in *mdx* mice

**Fig. 4.**
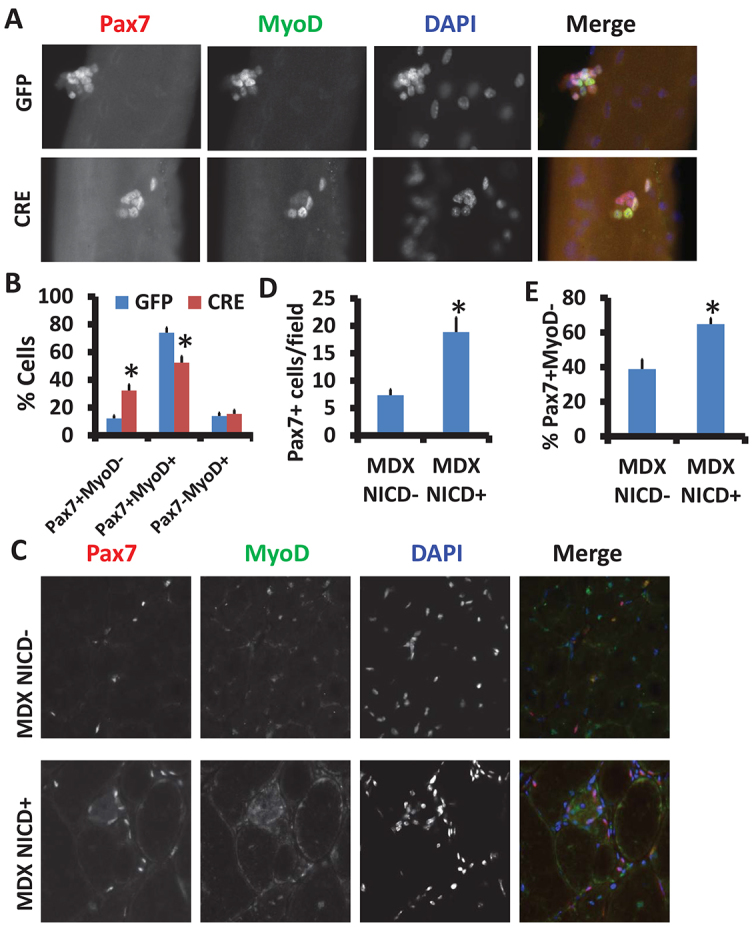
**Activation of Notch signaling promotes *mdx* satellite cell self-renewal.** (A) Muscle fibers from *Rosa26^NICD^/mdx* mice were cultured for 48 hours and infected by adenovirus (-GFP or -Cre) for a further 24 hours. Representative images of cell clusters labeled with Pax7 (red) and MyoD (green). Nuclei were counterstained with DAPI (blue). (B) Percentages of self-renewing (Pax7^+^MyoD^−^), proliferating (Pax7^+^MyoD^+^) and differentiating (Pax7^−^MyoD^+^) cells are shown (mean±s.e.m., *n*=3). (C) Two-month-old littermate control *mdx*/NICD^−^ (Pax7^CreER^/*mdx*) and *mdx*/NICD^+^ (Pax7^CreER/+^/Rosa26^NICD^/mdx) mice were injected intraperitoneally with tamoxifen for 5 consecutive days. Then TA muscles were injected with CTX and samples were collected 7 days after CTX injection. Representative images of TA cross sections stained with Pax7 (red) and MyoD (green) are shown. Nuclei were counterstained with DAPI (blue). (D) Number of Pax7^+^ satellite cells per field (mean±s.e.m., *n*=3). (E) Percentage of self-renewing (Pax7^+^/MyoD^−^) satellite cells. Data are mean±s.e.m. from three independent experiments. *P<0.05 compared with control.

To directly address whether reduced Notch signaling in *mdx* satellite cells is responsible for their self-renewal defects, we carried out gain-of-function studies using Cre/LoxP-mediated conditional gene expression tools. We first cultured EDL myofibers from *Rosa26^NICD^/mdx* mice, and used the adenovirus-Cre to activate NICD expression during the culture. After 72 hours of culture, myofibers were stained with Pax7 and MyoD (Fig. 4A). Quantitative analysis indicates that the percentage of Pax7^+^MyoD^−^ (self-renewing) satellite cells was drastically increased (from 12% to 32%) after Cre-induced Notch activation, compared to the control adenovirus-GFP treatment (Fig. 4B). Meanwhile the proportion of proliferating (Pax7^+^MyoD^+^) cells was significantly decreased by Notch activation (Fig. 4B). These results demonstrate that Notch activation improves self-renewal and inhibits the proliferation of satellite cells in *mdx* mice.

We next attempted to activate Notch signaling in *mdx* satellite cells *in vivo*. To do this, we established the *Pax7^CreER^/Rosa26^NICD^/mdx* triple transgenic mouse in which Notch signaling is specifically activated in *mdx* satellite cells upon tamoxifen induction. After five doses of tamoxifen (by i.p. injection), NICD (revealed by nuclear GFP expression as the *Rosa26^NICD^* mice also express nuclear GFP upon Cre activation) was specifically activated in the Pax7^+^ satellite cells (supplementary material Fig. S3A). Analysis of TA muscle cross sections (Fig. 4C) indicates that the number of Pax7^+^ satellite cells was increased significantly after Notch activation (Fig. 4D). More strikingly, the percentage of Pax7^+^MyoD^−^ (self-renewing) satellite cells was also significantly increased (from 40% to 60%) after NICD overexpression (Fig. 4E). These results provide compelling evidence that constitutive activation of Notch rescues the self-renewal defect of *mdx* satellite cells.

To examine how satellite-cell-specific stimulation of Notch signaling affects muscle pathology in *mdx* mice, we conducted histological analysis of TA muscle sections with and without CTX injury. Surprisingly, Notch activation in satellite cells failed to ameliorate muscle pathology or improve CTX-induced muscle regeneration in the *mdx* mice (supplementary material Fig. S3B). This was manifested by the reduced number of regenerated myofibers with an area of 500 μm^2^ or greater (supplementary material Fig. S3C). This observation is consistent with our recent report that constitutive Notch activation in satellite cells blocked muscle regeneration in WT mice (Wen et al., 2012) because of the well-known function of Notch signaling in inhibiting myogenic differentiation (Kopan et al., 1994). These data suggest that although constitutive Notch activation promotes self-renewal of satellite cells and increases satellite cell number in *mdx* mice, Notch signaling must be temporally suppressed during myogenic differentiation to allow proper progression of myogenesis.

## DISCUSSION

In this work, we identified that the satellite cells in *mdx* mice undergo drastic changes not only in the cell number but also in the cell activity. Specifically, the satellite cells in dystrophic muscles were defective in self-renewal capacity. In addition, we verified that the Notch signaling pathway was perturbed in *mdx* satellite cells using Notch reporter mice. Furthermore, we showed that constitutive activation of Notch signaling rescued the self-renewal deficiency of *mdx* satellite cells *in vitro* and *in vivo*. However, constitutive activation of Notch failed to improve muscle regeneration in *mdx* mice. Together, these results suggest that aberrant Notch signaling is responsible for the defective self-renewal capacity and that enhancement of Notch signaling leads to improved self-renewal of satellite cells in muscular dystrophy.

Muscular dystrophy has been characterized as an inherited disease featuring susceptibility to muscle damage and progressive muscle wasting. Given the indispensable role of satellite cells in muscle regeneration (Sambasivan et al., 2011a; von Maltzahn et al., 2013), the behavior of satellite cells, especially their replicative and differentiation capacity, determines the progression of muscle dystrophies. Few primary myoblasts can be isolated from DMD patients (Blau, 1983) and these myoblasts have reduced proliferative capacity (Lamperth et al., 1990). Correspondingly, primary myoblasts from *mdx* mice exhibit accelerated differentiation kinetics (Yablonka-Reuveni and Anderson, 2006), supporting the notion that the proliferative capacity is compromised in the satellite cells of dystrophic muscles. Our results that the satellite cell number and proliferative response to CTX stimulation declined with age in *mdx* mice provide direct *in vivo* evidence for age-dependent deficiencies in satellite cell activity.

Our findings that satellite cells in dystrophic muscles are defective in self-renewal capability are consistent with a previous study showing that muscle dystrophy results from an autonomous failure of satellite cells to maintain repetitive degeneration and regeneration cycles (Sacco et al., 2010). It is worth mentioning that murine somatic cells have longer telomeres compared to human cells, which significantly decreases the replicative senescence and increases the regenerative capacity of *mdx* satellite cells (compared to DMD satellite cells). To some extent, this explains why the *mdx* mice have a slower pathological progression relative to human DMD. However, our results indicate that the satellite cells in *mdx* mice still exhibit deficiencies in self-renewal capability. Specifically, *mdx* satellite cells cannot replenish themselves at all in 24-month-old mice, whereas the WT non-dystrophic satellite cells can still self-renew at this age (but at a significantly reduced rate compared to satellite cells from young animals). This finding prompted us to investigate the molecular mechanism behind the defective self-renewal capacity of *mdx* satellite cells. The mechanism governing the self-renewal of satellite cells is currently a topic of intense investigation. Recent studies have revealed a number of factors that induce the quiescence or self-renewal of satellite cells. These include the sprouty 1 (Shea et al., 2010), angiopoietin 1 (Ang1) and Tie2 pathway (Abou-Khalil et al., 2009), the Par-complex-dependent p38α and p38β MAPK pathway (Troy et al., 2012), microRNA-489 (Cheung et al., 2012) and nitric oxide (Buono et al., 2012). However, what signaling mechanism(s) regulate the self-renewal deficiency in *mdx* satellite cells has not been determined.

The Notch signaling pathway plays complicated and crucial roles in embryonic muscle development and postnatal myogenesis. Previous studies have demonstrated an interaction between dystrophin and the Notch pathway. In *Drosophila*, the membrane Dystrophin–Dystroglycan complex can interplay with the Notch ligand Delta (Kucherenko et al., 2008), which implies that the Notch signaling pathway might be perturbed in the absence of dystrophin in *mdx* muscles. This notion is supported by the microarray analysis of dystrophic muscles. A number of key genes involved in the Notch signaling pathway have been detected with altered levels in dystrophic muscles. Specifically, the Notch antagonist *Numb* is upregulated, whereas the Notch target genes *Hes1* and *Hey1* are downregulated drastically (Turk et al., 2005). Most recently, Church et al. have discovered that there is a reduction in the level of *Notch1* and *Hes1* mRNA in the TA muscles of *mdx* mice and DMD patients (Church et al., 2014). However, direct *in vivo* evidence demonstrating that an aberrant Notch signaling occurs in satellite cells in dystrophic muscles had been lacking. Using Cp-GFP as a reporter of the Notch signaling pathway activation, we now provide *in vivo* evidence that the Notch signaling pathway is perturbed in satellite cells of *mdx* muscles. This is the first direct *in vi*vo evidence that the Notch signaling pathway is inhibited in the satellite cells of dystrophic muscles. Our finding is consistent with the previous studies showing that Notch blockage in satellite cells can cause a muscular dystrophy phenotype as well as deterioration in muscle regeneration (Lin et al., 2013). In addition, Church et al. also show that Notch inhibition impedes the functional recovery of regenerated *mdx* muscles (Church et al., 2014). To rescue this self-renewal defect in dystrophic muscles, we established a mouse model to constitutively activate Notch signaling in satellite cells. As expected, Notch activation successfully ameliorated the self-renewal capacity, which corroborates the hypothesis that the impaired Notch signaling pathway contributes to the defective self-renewal capacity in the satellite cells of dystrophic muscles. Therefore, Notch activation might provide a potential route to prevent premature depletion of satellite cells in DMD patients. In addition, with respect to the long-term treatment of muscle dystrophy, our studies suggest that Notch inhibitor therapies might have potential side effects by accelerating the exhaustion of satellite cells. However, continuous Notch activation fails to improve regeneration of *mdx* muscles. We interpret this observation as indicative of a lack of myogenic differentiation due to the inhibition of MyoD and myogenin by Notch signaling. Taken together, a dynamic regulation of Notch signaling is necessary to balance self-renewal and differentiation of satellite cells in order to ameliorate the long-term regenerative defects of dystrophic muscles.

## MATERIALS AND METHODS

### Animals

*Myf5^nLacZ^* mice were provided by Shahragim Tajbakhsh (Pasteur Institute, Paris, France) (Christov et al., 2007). All other mice are available from Jackson Laboratories (ROSA26^N1ICD^, stock number 008159; *mdx*, stock number 001801; Pax7^CreER^, stock number 012476; and Cp-GFP, stock number 005854). Mice were maintained in a clean mouse facility at Purdue University. All procedures involving animal maintenance and experimental use were performed according to the guidelines presented by Purdue University’s Animal Care and Use Committee.

### Muscle injury and regeneration

Muscle regeneration was induced by cardiotoxin (CTX; Sigma-Aldrich, St Louis, MO) injection. Mice were first anesthetized using a ketamine-xylazine cocktail and then 50 μl of 10 mM CTX was injected into the tibialis anterior (TA) muscle. Muscles were harvested at 7 days post injection.

### Electroporation of DNA plasmids into TA muscles

About 10 μl of empty pEF-BOS Neo plasmid, Jag1 plasmid or RAMIC domain (NICD) plasmid were injected at a concentration of 0.5 μg/μl (in 0.9% NaCl) into TA muscles along the whole muscle length. Two spatula electrodes were then placed on each side of the muscle belly, and eight pulses (20 millisecond, 200 V/cm) at 1-second intervals were applied to the electrodes controlled by a BTX ECM 830 electroporator (Genetronics, San Diego, CA). The dosage was established in a preliminary study using the GFP plasmid, in which ~70% myofibers in the vicinity of the injection site and ~30% fibers in the whole muscle were GFP^+^ at 1 week after electroporation. Using this protocol, Jag1 or RAMIC was electroporated into the left TA muscles of the Cp-GFP mice, and the empty pEF-BOS vector was electroporated into the contralateral (right) TA as a control. At 1 week after electroporation, the TA muscles were harvested for assessment of Cp-GFP and Pax7 expression by immunohistochemistry.

### Isolation and culture of single fibers and primary myoblasts

Single myofibers were isolated from the extensor digitorum longus (EDL) muscles after collagenase A (Sigma) digestion for 45 minutes to 1 hour. Suspended fibers were collected and cultured in horse-serum-coated plates in Dulbecco’s modified Eagle’s medium (DMEM) supplemented with 10% fetal bovine serum (FBS; HyClone, Logan, UT), 2% chicken embryo extract (Accurate Chemical, Westbury, NY) and 1% penicillin-streptomycin for 72 hours.

Primary myoblasts were collected from limb skeletal muscle. These muscles were minced and digested with a cocktail of type I collagenase and Dispase B mixture. Debris was removed using filters from cells. Primary myoblasts were cultured in 100-mm collagen coated plates in the growth medium (F-10 Ham’s medium supplemented with 20% FBS, 4 ng/ml basic fibroblast growth factor and 1% penicillin-streptomycin) at 37°C under 5% CO_2_.

### Fluorescence-activated cell sorting

Cells were isolated from hindlimb muscles of 6- to 8-week-old mice. Erythrocytes were removed through the Red Blood Cell Lysing Buffer Hybri-Max (Sigma). Mononuclear cells were blocked with goat serum for 10 minutes and incubated with primary antibodies in DMEM with 2% FBS at 1×10^7^–3×10^7^ cell/ml for 15 minutes at 4°C. Cells were briefly washed and incubated with appropriate secondary antibodies (1:1000) at 4°C for 15 minutes. After staining, cells were washed, passed through 30-μm filters (Miltenyi Biotec) and suspended at a concentration of 1×10^7^ cells/ml. Cells were separated on a MoFlo cytometer (DakoCytomation) equipped with three lasers. Sorting gates were strictly defined based on single antibody-stained control cells as well as the forward and SSC patterns of satellite cells based on preliminary tests.

### Cryosection

Fresh TA muscles were embedded in optimal cutting temperature (OCT) compound (Sakura Finetek) and immediately frozen in dry-ice-cooled isopentane. Muscle blocks were cut to a 10-μm thickness with a Leica CM 1850 cryostat instrument. The sections were placed on Superfrost Plus glass slides (Electron Microscopy Sciences).

### Immunostaining and image capture

Muscle fibers and tissue sections were first fixed in 4% paraformaldehyde (PFA) and blocked in the blocking buffer containing PBS, 5% horse serum, 2% BSA, 0.2% Triton X-100 and 0.1% sodium azide for 60 minutes. Then the fibers and sections were incubated with primary antibodies (anti-Pax7, Developmental Studies Hybridoma Bank, University of Iowa; anti-MyoD, M-318, Santa Cruz Biotechnology; anti-β-galactosidase, MAB1802, Millipore Corporation; anti-MyoG, F5D, Santa Cruz Biotechnology; anti-GFP, ab13970, Abcam) diluted in blocking buffer overnight at 4°C, then incubated with secondary antibodies (goat anti-mouse Alexa Fluor 568, A21043, Invitrogen; goat anti-rabbit Alexa Fluor 488, #111-545-144, Jackson ImmunoResearch) and 4′,6-diamidino-2-phenylindole (DAPI) diluted in PBS for 30 minutes at room temperature and mounted with Dako fluorescent mounting medium (Glostrup, Denmark). Fluorescence pictures were taken with a Coolsnap HQ CCD camera (Photometrics, USA) driven by IP Lab software (Scanalytics, USA) in a Leica DMI 6000B fluorescence microscope (Mannheim, Germany). As the analysis of the immunofluorescence was qualitative, identical image handling and fluorescence scoring criteria were applied in all the experiments.

### Quantitative real-time polymerase chain reaction

RNA was extracted and purified from wild-type (WT) and *mdx* primary myoblast cell cultures using Trizol. Random hexamer primers were used for the reverse transcription from RNA to cDNA. The quantitative real-time polymerase chain reaction (qPCR) was performed with a Light Cycler 480 machine (Roche). The 18S-encoding gene was used as a housekeeping gene for normalization. For qPCR result analysis, the 2^−^^ΔΔct^ method was applied to calculate the fold change.

### Statistical analysis

The data are displayed as mean±s.e.m. *P*-values were calculated by a two-tailed Student’s *t*-test. *P*<0.05 was considered to be statistically significant.

## Supplementary Material

Supplementary Material
