# Supplementary material for: Notch signaling deficiency underlies age-dependent depletion of satellite cells in muscular dystrophy
Source: Dis Model Mech. 2014 Jun 6;7(8):997–1004. doi: 10.1242/dmm.015917 (PMC4107328; doi:10.1242/dmm.015917)
Supplement: Supplementary Material [file supp_7_8_997__index.html]

Notch signaling deficiency underlies age-dependent depletion of satellite cells in muscular dystrophy — Supplementary Material 

# Notch signaling deficiency underlies age-dependent depletion of satellite cells in muscular dystrophy

## DMM015917 Supplementary Material

**Files in this Data Supplement:**

- **Supplementary Material**
