# Supplementary material for: Notch signaling deficiency underlies age-dependent depletion of satellite cells in muscular dystrophy
Source: Dis Model Mech. 2014 Jun 6;7(8):997–1004. doi: 10.1242/dmm.015917 (PMC4107328; doi:10.1242/dmm.015917)
Supplement: Supplementary Material [file supp_7.8.997_DMM015917.pdf]

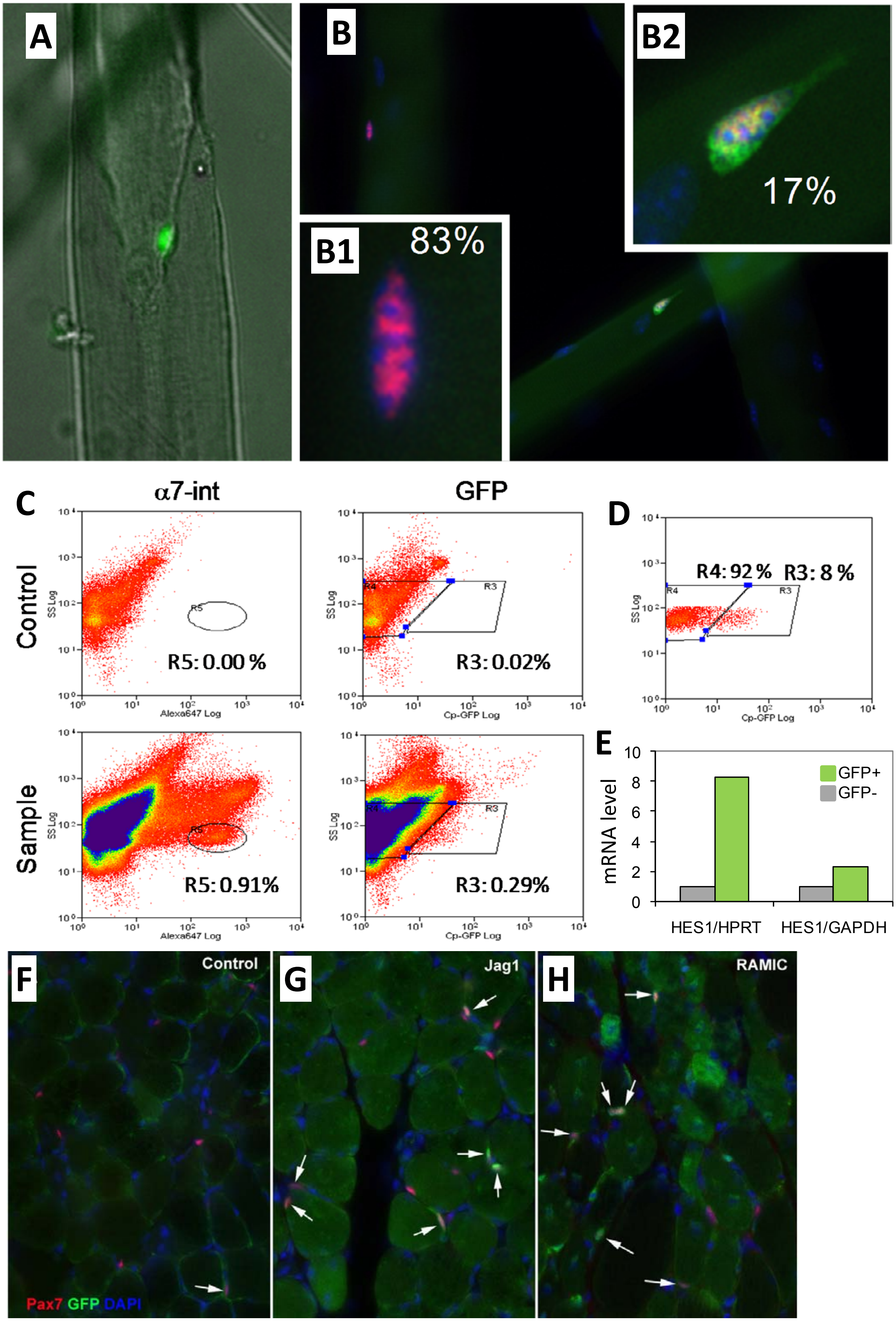
A
B
B2
17%
B1
83%
C
α7-int
GFP
Control
Sample
R5: 0.00 %
R3: 0.02%
R5: 0.91%
R3: 0.29%
R4
R3
R5
SS Log
Alexa647 Log
Cp-GFP Log
D
R4: 92 %
R3: 8 %
E
mRNA level
GFP+
GFP-
HES1/HPRT
HES1/GAPDH
F
Control
Pax7 GFP DAPI
G
Jag1
H
RAMIC

Fig. S1. The Cp-GFP transgenic mouse is a faithful reporter of Notch signaling in satellite cells. (A) GFP signal in a putative satellite cell on a freshly isolated live EDL myofiber. (B) Double labeling with Pax7 (Red, satellite cell marker) and GFP (Green) indicates that 83% satellite cells (Pax7$^+$) are GFP$^-$ (B1), but 17% are GFP+ (B2, n=102 cells counted from 3 mice). (C) Isolation of GFP$^+$ and GFP$^-$ satellite cells from the Cp-GFP mouse by fluorescent activated cell sorting using a7-integrin as a positive selection marker. (D) Analysis of sorted a7-integrin$^+$ cells indicates that 8% of cells are GFP$^+$. (E) Realtime PCR analysis indicate that sorted GFP$^+$ satellite cells expressed higher levels of *Hes1*, a canonical target of Notch signaling. (F-H) Activation of Notch signaling turns on Cp-GFP in satellite cells in vivo. Shown are images of Pax7 and GFP staining in control (F), Jag1 overexpressing (G), and RAMIC (NICD) overexpressing (H) TA muscle sections 7 days after electroporation of plasmids encoding empty vector, Jag1 cDNA and NICD cDNA, respectively. Arrows point to Pax7$^+$/GFP$^+$ satellite cells.

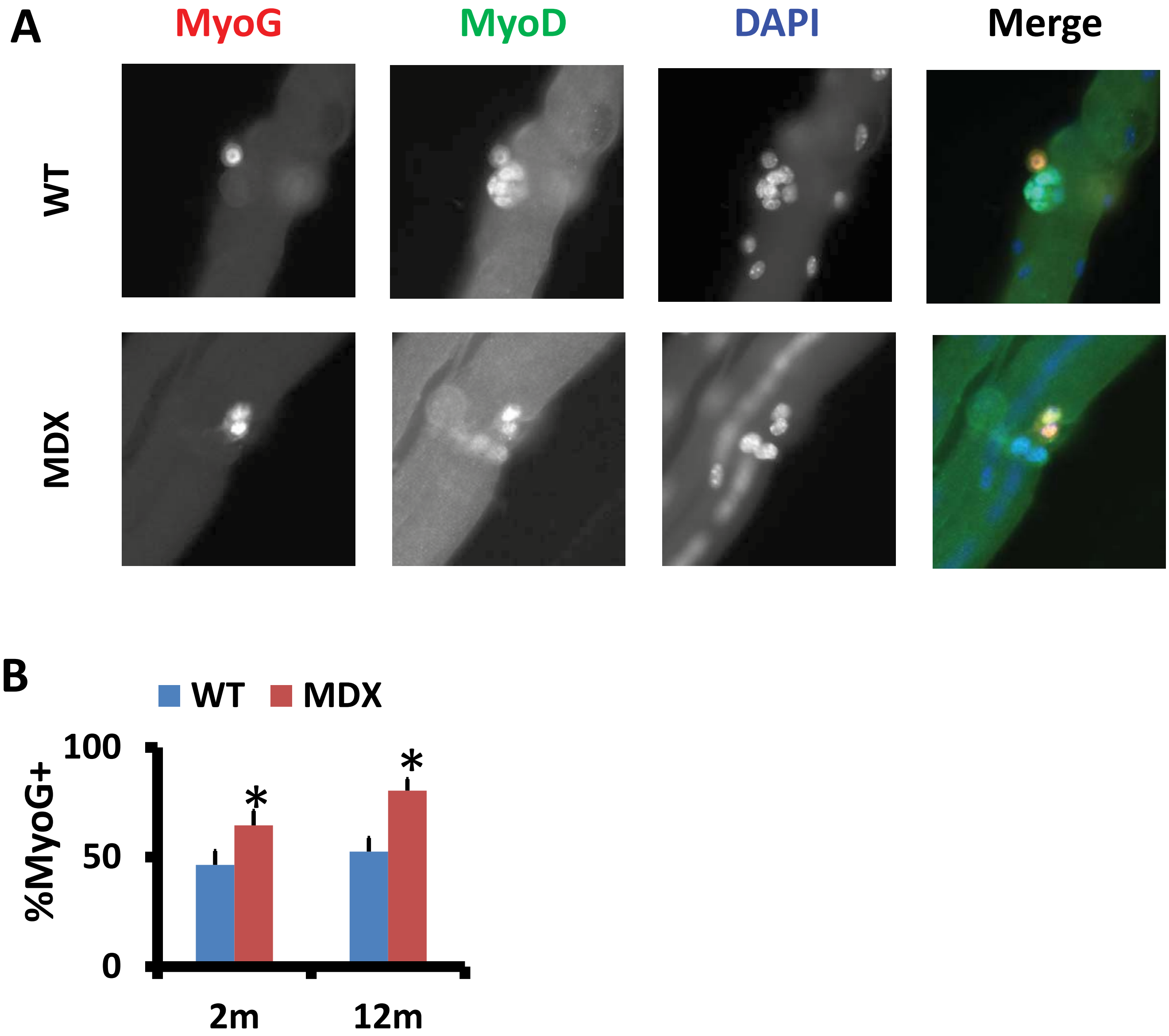

Fig. S2. *mdx* satellite cells have a higher tendency for terminal differentiation. (A) Representative images of satellite cell clusters from 2 and 12-months-old WT and *mdx* mice labeled with MyoG (red) and MyoD (green). Nuclei were counterstained with DAPI (blue). (B) Percentages of MyoG$^+$ cells per cluster at the indicated ages. Data were from 3 independent experiments with at least 20 clusters analyzed in each experiment. Error bars represent s.e.m. *P<0.05.

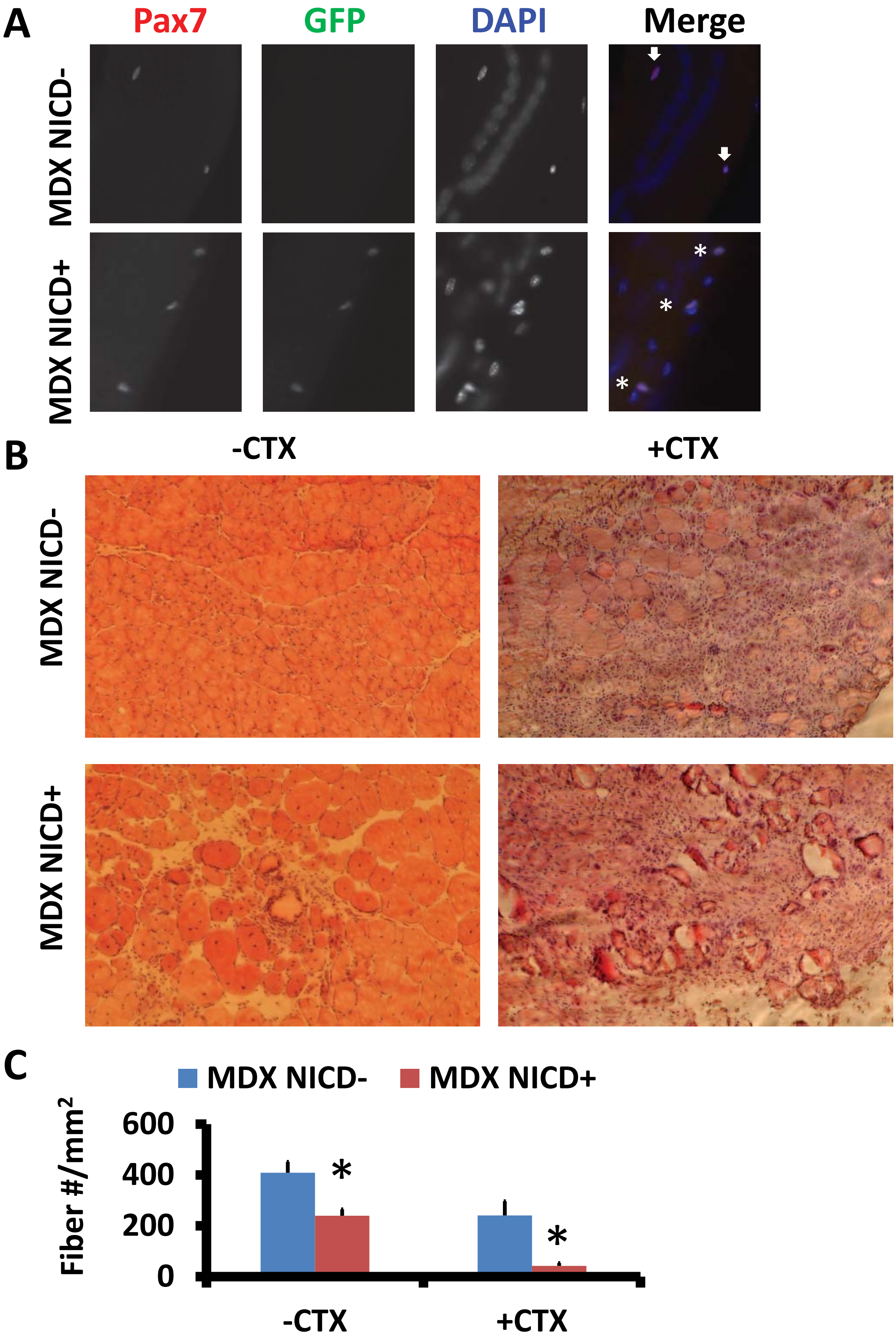

Fig. S3. Constitutive activation of Notch in *mdx* satellite cells fail to improve muscle pathology. (A) Representative images showing Notch pathway was activated in the *mdx*/NICD+ satellite cells after Tamoxifen injection. Muscle fibers from MDX/NICD- ($Pax7^{CreER/+}$/*mdx*) and MDX/NICD+ ($Pax7^{CreER/+}$/$Rosa26^{NICD}$/mdx) mice were labeled with Pax7 (red) and GFP (green). Nuclei were counterstained with DAPI (blue). GFP signal is turned on by Cre in the $Rosa26^{NICD\text{-}ires\text{-}nGFP}$ mice (called $Rosa26^{NICD}$ here). (B) Representative H&E staining images showing muscle morphology of resting and CTX-injured *mdx*/NICD- and *mdx*/NICD+ mice (refer to Fig. 4). (C) Quantitative analysis of number of myofibers (>500 μm^2) per unit field (1 mm^2) in resting (-CTX) and CTX-treated (+CTX) muscles. n=3.
